# Multi-Omics Blood Atlas Reveals Host Immune Response Features of Immunocompromised Populations Following SARS-CoV-2 Infection

**DOI:** 10.1016/j.mcpro.2025.101068

**Published:** 2025-09-10

**Authors:** Xiaodi Yang, Ye Shen, Bo Tang, Jialin Zhu, Bingjie Wang, Qingyun Wang, Wenmin Tian, Stefan Wuchty, Ziding Zhang, Zeyin Liang, Yujun Dong

**Affiliations:** 1 Department of Hematology, Peking University First Hospital, Beijing, China; 2 Center for Precision Medicine Multi-Omics Research, Institute of Advanced Clinical Medicine, Peking University, Beijing, China; 3 Department of Computer Science, University of Miami, Miami, Florida, USA; 4 Department of Biology, University of Miami, Miami, Florida, USA; 5 Institute of Data Science and Computation, University of Miami, Miami, Florida, USA; 6 Sylvester Comprehensive Cancer Center, University of Miami, Miami, Florida, USA; 7 College of Biological Sciences, China Agricultural University, Beijing, China

**Keywords:** SARS-CoV-2 infection, transcriptome, proteome, interactome, hematological malignancies

## Abstract

The dysregulation of human genes and proteins following SARS-CoV-2 infection significantly impacts the clinical symptoms and prognosis of COVID-19, particularly in immunocompromised individuals such as patients with hematological tumors. Despite this, a comprehensive multi-omics understanding of human host immune responses remains incomplete. Here, we conducted a multi-omics analysis of 89 peripheral blood samples (RNA sequencing) and 98 serum samples (proteome mass spectrometry) from 52 patients with COVID-19, including patients with hematological tumors and non-tumor individuals. By integrating transcriptomic, proteomic, and interactome data, we compared differentially expressed genes (DEGs) and proteins (DEPs) across infection stages and clinical outcomes to gain insights into the mechanisms of SARS-CoV-2 infection. Our analysis revealed distinct and overlapping transcriptomic and proteomic responses to SARS-CoV-2 infection. DEGs were predominantly associated with innate immune responses and viral processes, while DEPs were linked to actin cytoskeleton organization and protein kinase regulation. Notably, DEGs and DEPs often exhibited opposing regulatory patterns, suggesting post-transcriptional and post-translational mechanisms. Tumor patients showed more severe proteomic perturbations, with a higher proportion of DEPs functioning as key hub proteins in cellular networks. Network-based drug repositioning identified potential therapeutic targets, including HSPA8, SRC, STAT1, APOE, and APP. Clinical analysis indicated that patients with long COVID experienced more severe coagulation abnormalities, immunosuppression, and myocardial injury, while acutely deceased patients exhibited abnormally activated immune responses. Our study provides a comprehensive resource for understanding the molecular mechanisms of SARS-CoV-2 infection in patients with hematological tumors. By integrating multi-omics data, we highlight the importance of proteomic changes in disease progression and identify potential therapeutic targets for COVID-19 and long COVID.

## Highlights

• Multi-omics profiling reveals distinct immune dysregulation in patients with hematological tumors having COVID-19.
• Tumor patients exhibit severe proteomic network perturbations, where hub proteins show stronger dysregulation compared to non-tumor individuals.
• Both long COVID and fatal cases showed coagulation dysfunction and myocardial injury but divergent immune signatures (immunosuppression in long COVID versus hyperactivation in fatalities).
• Network-based drug repositioning uncovers potential therapeutic targets and the associated drug candidates.

## Graphical Abstract

**Figure:**
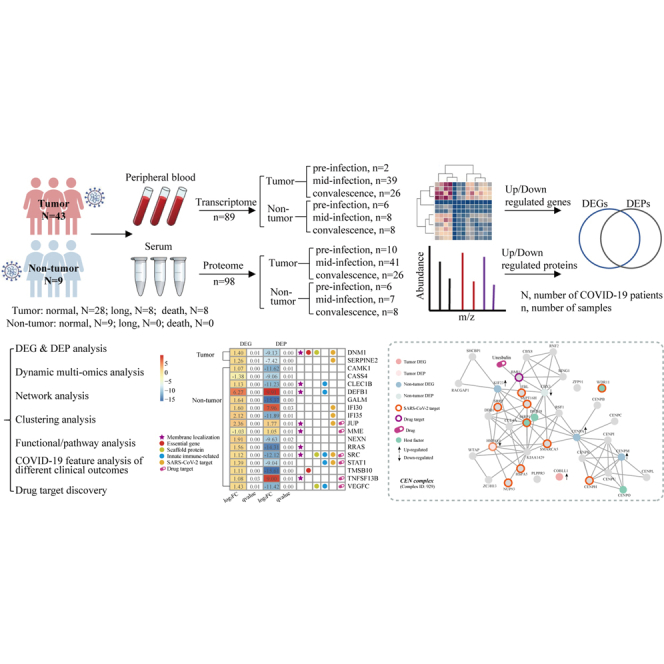

## In Brief

This multi-omics study reveals distinct immune dysregulation in hematological tumor patients with COVID-19, showing severe proteomic network perturbations and divergent immune signatures between long COVID (immunosuppression) and fatal cases (hyperactivation). Network analysis identifies potential therapeutic targets (*e.g.*, HSPA8, SRC, and STAT1) and drug candidates, offering insights into COVID-19 mechanisms and tailored treatment strategies for high-risk patients.

The coronavirus infectious disease 2019 (COVID-19), caused by SARS-CoV-2, remains a major public health threat as a consequence of its high infectivity and the emergence of new variants (https://data.who.int/dashboards/covid19/data). Despite the availability of several antiviral drugs and vaccines such as Paxlovid, Molnupiravir, and Azvudine, treating COVID-19 remains challenging (1, 2, 3, 4, 5), particularly in immunocompromised individuals such as patients with hematological malignancies (6, 7). These patients often exhibit weaker immune responses and reduced viral clearance capabilities, leading to higher risks of adverse outcomes and long-term sequelae (8, 9). Understanding the molecular mechanisms underlying SARS-CoV-2 infection in these vulnerable populations is critical for developing targeted therapies with more specific and definitive effects. While recent studies have advanced our knowledge of the host immune response to COVID-19 (10, 11, 12, 13), a comprehensive comparative analysis of transcriptomic and proteomic profiles in patients with and without hematological tumors remains limited. Such analyses are essential for identifying differences in immune regulation and uncovering potential therapeutic targets.

Transcriptome, proteome, and interactome analyses are powerful omics technologies that can uncover the mechanisms underlying SARS-CoV-2 infection and host immune responses (14, 15, 16, 17, 18, 19, 20). A systematic comparison of transcriptomic and proteomic profiles in COVID-19 patients with and without hematological tumors, or among hematological tumor patients with different clinical outcomes, is essential for understanding the molecular differences in gene regulation and protein expression during infection. Critically, pairing whole blood transcriptomics (reflecting leukocyte-specific responses) with serum proteomics (capturing systemic inflammation/tissue damage signals) provides complementary insights into immune-vascular crosstalk—a key determinant of COVID-19 severity in immunocompromised hosts (21, 22). Here, we analyzed transcriptomic and proteomic data from a cohort of 187 samples, including patients having COVID-19 with and without hematological tumors. By integrating RNA sequencing, proteome mass spectrometry, and interactome data, we aimed to provide a comprehensive understanding of host-virus interactions at the molecular level. Specifically, we focused on the human-SARS-CoV-2 protein interactome, which plays a pivotal role in viral pathogenesis. While numerous protein–protein interactions (PPIs) between the human host and SARS-CoV-2 have been identified, the role of host protein complexes in viral infection is often overlooked. Viruses frequently exploit these complexes for replication (23, 24), hijacking host cellular processes to facilitate their survival and propagation (25, 26). To address this gap, we integrated human-SARS-CoV-2 interaction data with human protein complex information, enabling a deeper analysis of SARS-CoV-2 infection and host immune responses at the interactome and protein complex levels.

Our systematic investigation revealed significant modulation of genes and proteins in whole blood and serum, highlighting both commonalities and differences in transcriptomic and proteomic responses to SARS-CoV-2 infection. Integrative analysis uncovered evidence of immune dysregulation leading to vascular, renal, and cardiac muscle injuries. Notably, patients having long COVID with hematological tumors exhibited more severe immunosuppression and myocardial injury, while acutely deceased patients showed activated immune responses accompanied by myocardial injury compared to those with normal infections. Furthermore, through network-based drug repositioning, we identified potential therapeutic targets for patients having COVID-19 with hematological tumors across various clinical outcomes. This study underscores the importance of integrating transcriptomic and proteomic data to enhance our understanding of COVID-19 pathogenesis. By revealing the molecular mechanisms of immune dysregulation in hematological tumor patients, our work provides a valuable resource for developing targeted therapies and improving clinical outcomes in this vulnerable population.

Previous large-scale proteomic studies have illuminated systemic host responses to COVID-19. For instance, Ahern *et al*. (27) identified inflammation and coagulation markers as hallmarks of severe infection, while Filbin *et al*. (28) linked endothelial injury proteins to long-term sequelae. However, these studies predominantly focused on general populations or solid tumors, leaving a critical gap in understanding immunocompromised hosts like hematological tumor patients. Such patients exhibit impaired viral clearance and altered immune cell function (29), suggesting potentially unique proteomic dynamics. Our work addresses this gap by integrating transcriptomic and proteomic profiling specifically in hematological malignancies, enabling a systems-level view of immune dysregulation inaccessible via single-omics approaches.

## Experimental Procedures

### Study Design and Participants

#### Cohort Characteristics

**Fig. 1:**
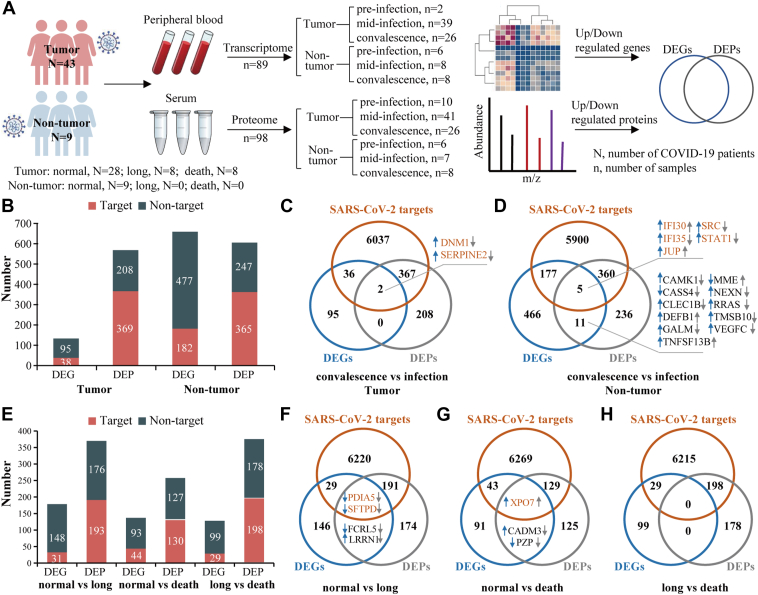
**Multi-omics analysis of COVID-19 patients with and without hematological tumors.***A*, Schematic of the overall experimental workflow of the sample collection, quantitative transcriptome/proteome profiling, and bioinformatics analysis. *B*, identified targeted and non-targeted differentially expressed genes and proteins of COVID-19 patients with and without tumors post-SARS-CoV-2 infection. *C* and *D*, Venn diagrams showing the overlap of differentially expressed genes and proteins of COVID-19 patients with and without tumors post-SARS-CoV-2 infection, respectively. *E*, identified targeted and non-targeted differentially expressed genes and proteins of COVID-19 patients with tumors grouped by clinical outcomes (normal infection, long infection, acute death) during the acute infection phase (mid-infection). *F–H*, Venn diagrams showing overlap of differentially expressed genes and proteins of COVID-19 patients with tumors in “normal infection *versus* long infection”, “normal infection *versus* acute death”, and “long infection *versus* acute death” groups, respectively. *Blue* and *gray* circles/rectangles indicate differentially expressed genes and proteins, respectively. *Yellow circles/rectangles* indicate that genes/proteins, i.e., SARS-CoV-2 targets, interact with SARS-CoV-2 proteins. *Blue* and *gray* up/down arrows indicate up-/downregulation of the differentially expressed genes and proteins, respectively.

We recruited 52 patients with COVID-19 from the Department of Hematology at Peking University First Hospital (December 2022–April 2023) (Fig. 1*A*). The cohort comprised 43 inpatients with hematological malignancies and 9 non-tumor healthy staff members without comorbidities from the same department. Among the hematological tumor patients, a subset received individualized antiviral regimens (Supplemental Table S1), whereas all non-tumor participants received no antiviral treatment. This may introduce potential selection biases such as age and comorbidity gap. Such demographic imbalance could lead to overestimation of immune response differences between groups, as age and comorbidities are established confounders in COVID-19 outcomes. Diagnosis of COVID-19 was confirmed by SARS-CoV-2 nucleic acid testing (RT-PCR) from pharyngeal swabs. Participants with hematological tumors were stratified into three groups based on clinical outcomes: i) normal infection (n = 28) defined by complete symptom resolution (including fever, cough, and other COVID-19-related manifestations) coupled with viral clearance (negative RT-PCR within 14 days post-diagnosis); ii) persistent long infection (n = 8) characterized by sustained viral positivity (RT-PCR+) and symptom duration exceeding 4 weeks post-infection, following long COVID criteria of United Kingdom National Institute for Health and Care Excellence guidelines (30, 31); iii) acute death (n = 8) defined as occurring within 14 days post-infection. Non-tumor individuals (n = 9) were met with normal infection criteria.

#### Sample Collection

A total of 89 peripheral whole blood samples and 98 serum samples were collected for transcriptome sequencing and proteome mass spectrometry (MS), respectively (Fig. 1*A*). Peripheral whole blood samples were collected via standard venipuncture in 4 ml EDTA-anticoagulated violet-top vacuum tubes. The samples were centrifuged at 1500*g* for 10 min to separate serum for proteomic analysis. The remaining whole blood was mixed with TRIzol for transcriptomic analysis. Both whole blood and serum samples were frozen at −80 °C until further processing, with storage duration not exceeding 12 months. Samples were aliquoted to ensure single-use without freeze-thaw cycles. Identical processing protocols were applied across all specimens. Peripheral whole blood samples were categorized based on the timeline of SARS-CoV-2 infection: pre-infection (n = 8), mid-infection (n = 47), and post-infection convalescence (n = 34). Serum samples were categorized based on the timeline of SARS-CoV-2 infection: pre-infection (n = 16), mid-infection (n = 48), and post-infection convalescence (n = 34). Mid-infection samples refer to the acute phase of infection, collected within 3 days after symptom onset when patients tested positive by nucleic acid testing (NAT) of pharyngeal swabs. The convalescence samples were collected approximately 2 to 3 months after recovery, defined as absence of symptoms plus confirmed negativity by SARS-CoV-2 NAT. Convalescence sampling was contingent on clinical recovery (symptom resolution + viral clearance). Acute death patients had no recovery phase, while long COVID patients remained infected at sampling. Pre-infection samples were limited by rapid pandemic exposure in this immunocompromised cohort. Paired mid-infection/convalescence samples enable longitudinal analysis of host responses. Clinical data including sex, age, diagnosis, symptoms, complications, antiviral treatment, and biomedical parameters at different COVID-19 stages were collected from all patients with hematological tumors (Table 1 and Supplemental Table S1).

**Table 1:** Overall clinical characterization comparison of COVID-19 patients with hematological tumors in different infectious groups

Group	Normal (n = 28)	Long (n = 8)	Death (n = 8)	*p* value^1^	*p* value^2^	*p* value^3^
Age	23–74 (51)	21–78 (52)	23–78 (59)	0.790	0.171	0.494
Sex				1.000	0.424	0.608
Male	15	4	6			
Female	13	4	2			
Complication	18	4	7	0.683	0.388	0.282
Anti-viral treatment	11	8	7	**0.003**	**0.008**	1.000
WBC	0.3–35.7 (5.56)	0.8–36.6 (8.03)	0.8–8.6 (4.53)	1.000	0.939	0.916
NE	0.1–7.4 (2.87)	0.7–12.4 (4.30)	0.4–92 (13.78)	0.246	0.662	0.916
LY	0.1–4.52 (1.01)	0.1–23.3 (3.33)	0.1–1.2 (0.50)	0.194	0.141	0.833
HGB	54–151 (96.32)	37–135 (99.88)	65–136 (91)	0.402	0.458	0.318
PLT	13–354 (133.32)	75–661 (199.88)	16–212 (78.63)	0.435	0.110	**0.036**
CRP	0.68–158.89 (35.45)	1.26–142 (36.42)	3.91–142 (60.70)	0.952	0.168	0.297
IL6	1.5–326.46 (34.38)	1.22–117.31 (27.08)	8.82–428.93 (87.76)	0.981	**0.018**	0.132
PCT	0.02–0.63 (0.17)	0.02–4.02 (0.66)	0.11–8.01 (2.11)	0.791	**0.002**	**0.032**
ALB	25.8–46.8 (36.50)	24.5–45.2 (34.08)	24.5–34.8 (30.95)	0.402	**0.011**	0.248
PT	9.1–17 (11.72)	10.5–14.2 (12.23)	11.2–17.3 (14.04)	0.302	**0.020**	0.110
INR	0.8–1.46 (1.01)	0.92–1.23 (1.06)	0.97–1.49 (1.22)	0.292	**0.020**	0.110
APTT	0.89–43.6 (29.14)	23.1–36.9 (30.67)	27.1–43 (32.67)	0.578	0.312	0.406
Fibrinogen	2.16–6.27 (3.74)	1.45–4.49 (3.38)	2.29–6.38 (3.72)	0.805	0.695	0.654
D-Dimer	0.06–15.65 (1.11)	0.08–0.58 (0.27)	0.1–5.74 (1.24)	0.210	0.710	0.200
FDP	1–114.5 (9.06)	1.1–4.93 (2.26)	1–36.24 (8.72)	0.208	0.788	0.404
TT	12.1–27.6 (15.11)	13.9–20.1 (16.3)	12.9–19.5 (15.66)	**0.050**	0.457	0.563

*p* value^1^, Normal *versus* Long; *p* value^2^, Normal *versus* Death; *p* value^3^, Long *versus* Death; The bold *p* values represent statistical significance.

WBC, white blood cell; NE, neutrophilic granulocyte; LY, lymphocytes; HGB, hemoglobin; PLT, platelets; CRP, C-reactive protein; IL6, interleukin 6; PCT, procalcitonin; ALB, albumin; PT, prothrombin time; INR, international normalized ratio; APTT, activated partial thromboplastin time; FDP, fibrinogen degradation products; TT, thrombin time.

#### Ethics Approval

Our study strictly adhered to the principles of the Declaration of Helsinki. Written informed consent was obtained from all participants, and the study (ClinicalTrials.gov, NCT05683353) was approved by the Biomedical Research Ethics Committee of Peking University First Hospital.

### Transcriptomic Profiling

#### RNA Sequencing

Peripheral whole blood samples were collected in TRIzol and stored at −80 °C until RNA extraction. Total RNA was extracted using the Standard Sensitivity RNA Analysis Kit (Qiagen) with globin mRNA depletion. RNA integrity was verified (RIN ≥7) using Agilent 2100 Bioanalyzer. Sequencing libraries were prepared using the NEBNext Ultra RNA Library Prep Kit following the manufacturer's protocol. 100 bp paired-end sequencing was performed on BGI platform. Detailed protocols are available in our previous work (32).

#### RNA-Seq Data Analysis

Raw sequencing data were processed as follows. Quality control: adapter sequences and low-quality reads were removed using SOAPnuke (33). Alignment: clean reads were mapped to the human reference genome (GRCh38) using HISAT2 (34). Quantification: transcript assembly and gene expression quantification were performed using StringTie (35) and RSEM (36), with normalization to transcripts per million (TPM). Transcriptomic data were derived from our prior study (32), using the identical patient cohort for which new proteomic data were generated herein. This paired design ensures direct biological comparability between molecular layers within the same individuals. Detailed RNA sequencing methodology follows (32).

### Proteomic Profiling

#### Depletion of Highly Abundant Proteins From Serum Samples

Highly abundant proteins were depleted from serum using the “Multi Affinity Removal Column, Human-14” (Agilent Technologies). Briefly, 20 μl of serum was mixed with 60 μl of buffer A, transferred to a Spin-X 0.22 μm cellulose acetate filter (COSTOR), and centrifuged at 16,000*g* for 1 min. The filtrate was processed using an Agilent 1290 liquid chromatography system. Protein concentrations of the depleted samples were determined using a BCA Protein Assay Kit (Thermo Scientific).

#### Protein Digestion and Peptide Preparation

A 50 μg aliquot of the depleted serum was precipitated overnight at 4 °C with 1/3 volume of trichloroacetic acid (TCA) solution, followed by centrifugation at 16,000*g* for 30 min at 4 °C. The precipitate was washed three times with acetone and dried using a vacuum concentrator (Labconco). The dried precipitate was dissolved in 40 μl of 8 M urea (pH 8.5), reduced with 20 mM (2-carboxyethyl) phosphine hydrochloride (TCEP), and alkylated with 40 mM iodoacetamide (IAA). The mixture was diluted with 200 μl of 100 mM Tris-HCl buffer (pH 8.5) to reduce the urea concentration to 1.3 M and digested with 3 μg of trypsin at 37 °C for 16 h. Peptides were desalted using a Monospin C18 column (GL Science, Tokyo, Japan), dried, and reconstituted in Milli-Q water with 0.1% formic acid for liquid chromatograph-mass spectrometer (LC-MS) analysis. Indexed retention time (IRT) calibration peptides were added prior to data-independent acquisition (DIA).

#### Sample Preparation for Spectral Library Construction

After the samples were prepared as peptides, 2 μg of peptides of each sample was taken and mixed. For spectral library construction, approximately 70 μg of mixed peptides were fractionated using a high-pH reversed-phase HPLC system (1290 Infinity, Agilent). Briefly, the mixed peptides were dissolved in buffer A (20 mM ammonia, 2% acetonitrile, pH 10.5) and loaded onto an Accucore C18 column (2.1 mm × 150 mm, 2.6 μm particles; Thermo Fisher Scientific). Separation was performed using a 56-min linear gradient from 5% to 80% buffer B (20 mM ammonia, 98% acetonitrile, pH 10.5) at a flow rate of 0.4 ml/min. 60 fractions were collected and concatenated into 20 pooled fractions by combining every 20th fraction (*e.g.*, 1, 21, 41, etc). The pooled fractions were dried by vacuum centrifugation, redissolved in 10 μl of Milli-Q water with 0.1% formic acid, and spiked with IRT peptides for chromatographic correction.

#### LC-MS/MS Analysis

The LC-MS/MS analysis was performed using an EASY-nLC 1200 HPLC system coupled to a Q Exactive HF-X mass spectrometer (Thermo Scientific). Peptides were separated on a reversed-phase C18 column (25 cm × 75 μm, 1.7 μm; IonOpticks) over 90 min. The elution gradient for solvent B (0.1% formic acid in 80% acetonitrile) was from 9% to 95%. The flow rate was set at 300 nl/min at 50 °C.

The mass spectrometer was operated in positive mode using either data-dependent acquisition (DDA) or DIA.

#### DDA and DIA Modes

For DDA mode, the top 20 precursor ions were selected for fragmentation. Full MS1 scans were acquired at a resolution of 60,000 (scan range: 350–1200 m/z) with a target automatic gain control (AGC) value of 3 × 10^6^ and a maximum injection time of 50 ms. MS2 scans were acquired at a resolution of 15,000 (scan range: 200–2000 m/z), with a target AGC value of 5 × 10^5^ and a maximum injection time of 120 ms. The normalized collisional energy (NCE) was set to 30, the isolation window was set to 1.6 m/z, and the ion selection threshold was set to 6.7 × 10^4^. Dynamic exclusion was applied for 30 s. For single-shot proteomics with DIA, MS1 scans were acquired at a resolution of 60,000 (scan range: 350–1200 m/z), with a target AGC value of 3 × 10^6^ and a maximum injection time of 50 ms. Precursors were isolated with 60 dynamic non-overlapping windows covering 350 to 1200 m/z and fragmented with an NCE of 28. The width of the windows is shown in Supplemental Table S2. The cycle time is 0.07 s. MS2 scans were acquired at a resolution of 30,000, with a target AGC value of 1 × 10^6^ and automatic maximum injection time settings.

#### Spectral Library Generation and Database Search

High-confidence MS/MS Spectral libraries were generated from DDA raw files using the “Library → Generate Spectral Library” module (Library workflow) in Spectronaut version 16.0 (Biognosys). Subsequently, DIA data analysis was performed using the “Analysis → Set Up a DIA Analysis” module (Analysis workflow). Spectral Library Generation and DIA data analysis shared the same parameter settings, differing only in their respective processing modules. All searches were performed against the human UniProt database (20,421 entries, downloaded in July 2019). Specifically, searches used carbamidomethylation as a fixed modification, with acetylation of the protein N-terminus and oxidation of methionines as variable modifications. The mass tolerance for precursor ions was set to 10 ppm, and for fragment ions to 20 ppm. Trypsin/P proteolytic cleavage rule was used, permitting a maximum of two missed cleavages, a minimum peptide length of 7 amino acids, and a maximum peptide length of 52 amino acids. The false discovery rate (FDR) thresholds for library generation and DIA analyses were both set to 0.01. All peptides included in the spectral library have been listed in Supplemental Table S3. All raw MS data have been deposited in Supplemental Table S4. Protein identification confidence was ensured by applying a strict filtering threshold: only proteins supported by ≥ 2 unique peptides were retained for downstream analysis. Identifications based on a single unique peptide were excluded to guarantee high-confidence results.

### Differential Expression Analysis and Statistical Rationale

We performed comprehensive differential expression analyses for both proteomic and transcriptomic data. For differentially expressed proteins (DEPs) identification, we compared protein expression between mid-infection and convalescence samples. Raw proteomic intensities were preprocessed using OmicsBean, where missing values (zeros) were imputed via the DBB imputation algorithm, followed by quantile normalization to generate normalized protein expression (NPX) values. Differential expression analysis was then performed by calculating fold-change values using Genefilter and conducting two-sided Student's t-tests, with Benjamini-Hochberg (BH) FDR multiple testing corrections. Proteins with |log_2_ fold change| > 1 and BH-adjusted *p*-value <0.05 were defined as DEPs. For differentially expressed genes (DEGs) analysis, RNA-Seq data were processed using DESeq2 (v1.34.0) (37), which employs a negative binomial generalized linear model and Wald tests for significance assessment, followed by BH correction for FDR control. Genes with |log_2_ fold change| > 1 and BH-adjusted *p*-value <0.05 were identified as DEGs. These consistent thresholds (|log_2_ fold change| > 1, BH-adjusted *p*-value <0.05) were chosen to ensure biological relevance by excluding minor fluctuations, maintain comparability with established omics studies, and optimize the balance between detection sensitivity and specificity.

Transcriptomic profiling of whole blood samples captures RNA signatures from circulating immune and non-immune cells, reflecting cellular-level processes. In contrast, serum proteomic analysis detects proteins originating from diverse tissues (*e.g.*, vascular endothelium, platelets, cardiac muscle), representing systemic/tissue-level signals. Integrating these complementary layers enables multi-scale systems analysis across cellular, tissue, and organ hierarchies, as established in prior clinical immunology studies (38, 39, 40, 41, 42, 43). This approach tests our hypothesis that immune cell dysregulation contributes to coagulation dysfunction and tissue injury in immunocompromised hosts.

### Network and Functional Analysis

#### PPI Network Construction

We initially collected experimentally determined human-SARS-CoV-2 PPIs and human PPIs from BioGRID (44). After eliminating redundant and non-physical interactions, we obtained 23,871 human-SARS-CoV-2 PPIs covering 6443 human proteins (also called SARS-CoV-2 targets) and 31 SARS-CoV-2 proteins, and 80,258 human PPIs involving 11,141 human proteins. Additionally, we collected 5204 and 6965 human protein complexes from CORUM (23) and hu.MAP (24), respectively. We mapped human PPIs onto these complexes, removed those without any human PPIs and eliminated redundant complexes with the same subunits, resulting in a total of 9475 human protein complexes.

#### Topological and Functional Annotation

To elucidate network patterns of DEGs and DEPs post-SARS-CoV-2 infection, we performed centrality analysis by calculating the number of interaction partners (i.e., degree) of DEGs/DEPs in the human PPI network using the R package “igraph”. To characterize the functional roles of DEGs and DEPs in hematological tumor patients and non-tumor individuals, we focused on four categories of functional genes/proteins capturing essential genes, scaffold proteins, SARS-CoV-2's host factors, and human innate immune-related proteins. We collected 5169 human essential genes indispensable for various cellular processes (45, 46), 273 scaffold proteins from ScaPD (47), 299 SARS-CoV-2's host factors from large-scale CRISPR screening studies (48, 49, 50), and 1044 human innate immune-related proteins from InnateDB (51). Scaffold proteins are crucial in cellular signaling pathways as a consequence of their role in complex assembly. Host factors include dependency factors and restriction factors, which facilitate or inhibit viral infection, respectively. Innate immune-related proteins are essential for the initial defense against viral invasion and the regulation of adaptive immunity responses.

#### Network Module Identification

To analyze targeted DEGs and DEPs, we constructed comprehensive interaction networks integrating human-SARS-CoV-2 PPIs and human PPIs using Cytoscape (52). Human PPIs involving these DEGs/DEPs were further analyzed to identify key human genes/proteins/targets and their associated pathways. Densely interconnected human protein clusters, representing potential functional modules, were detected with the MCODE plugin (53). The resulting modules were then functionally annotated and visualized to highlight critical interaction networks.

#### Functional and Pathway Enrichment Analysis

As for functional and pathway enrichment analysis of the identified or targeted DEGs/DEPs/modules, we obtained Gene Ontology (GO) annotation data from http://current.geneontology.org/ (54) and KEGG pathway data from https://www.genome.jp/kegg/ (55). Enriched GO terms and KEGG pathways were determined by hypergeometric tests with a cutoff of Benjamin-Hochberg corrected *p*-value <0.05. For transcriptomics (DEG) analysis, all human proteins mapped to Cellular Component, Biological Process, and Molecular Function ontologies, as well as all human proteins in KEGG pathways served as reference background. For serum proteomics (DEP) analysis, to accurately reflect the blood-specific proteome, the reference background comprised the 4294 human blood-circulating proteins with experimental evidence in the Human Protein Atlas (HPA) database (https://www.proteinatlas.org/) (56), mapped to the respective GO ontologies and KEGG pathways.

#### Drug Repositioning

To identify potential drug targets and repositioning opportunities, we retrieved drug target information, drug details, drug-target associations, and drug-disease relationships from the Therapeutic Target Database (TTD) (57). Furthermore, we mapped them onto our identified protein complex subnetworks and network modules for subsequent drug repositioning analysis.

## Results

### Comparative Landscape of DEGs and DEPs Post SARS-CoV-2 Infection

Overall, RNA sequencing and MS analysis identified 18,719 genes, 28,474 peptides, and 2021 proteins across all samples (Supplemental Tables S4 and S5). By integrating transcriptomic and proteomic data, we detected 133 DEGs and 577 DEPs in tumor patients, and 659 DEGs and 612 DEPs in non-tumor individuals, comparing post-infection convalescence and mid-infection phases (Fig. 1*B*). Among these, 38 DEGs/369 DEPs in tumor patients and 182 DEGs/365 DEPs in non-tumor individuals interacted with SARS-CoV-2 proteins (Fig. 1*B*). While few overlaps were observed between DEGs and DEPs in both groups, some genes and proteins were regulated in opposite directions, highlighting disparities between transcriptional and translational regulation (Fig. 1, *C* and *D*). Further analysis of tumor patients revealed distinct DEGs and DEPs across infection statuses (normal, long, and acute death), with several overlaps in “normal *versus* long” and “normal *versus* death” groups (Fig. 1, *E*–*H*). Non-tumor individuals exclusively presented normal infection outcomes, while tumor patients showed heterogeneous severity (long COVID and death). This aligns with epidemiological reports of elevated complication risks in immunocompromised hosts (6, 7), but direct group comparisons require caution due to differential baseline vulnerability. To evaluate the variability by comparing DEGs and DEPs across biological replicates, we conducted correlation and principal component analysis (PCA) on DEGs and DEPs of all samples. Specifically, we calculated Pearson correlation coefficients for both DEGs and DEPs across biological replicates. The high mean correlation coefficients (DEGs: r > 0.85; DEPs: r > 0.78) confirm strong consistency between replicates (Supplemental Fig. S1, *A* and *B*). PCA demonstrated that between-group variation exceeds within-group variation, further supporting the robustness of our dataset (Supplemental Fig. S1, *C* and *D*).

### Functional Enrichment Analysis of DEGs and DEPs

**Fig. 2:**
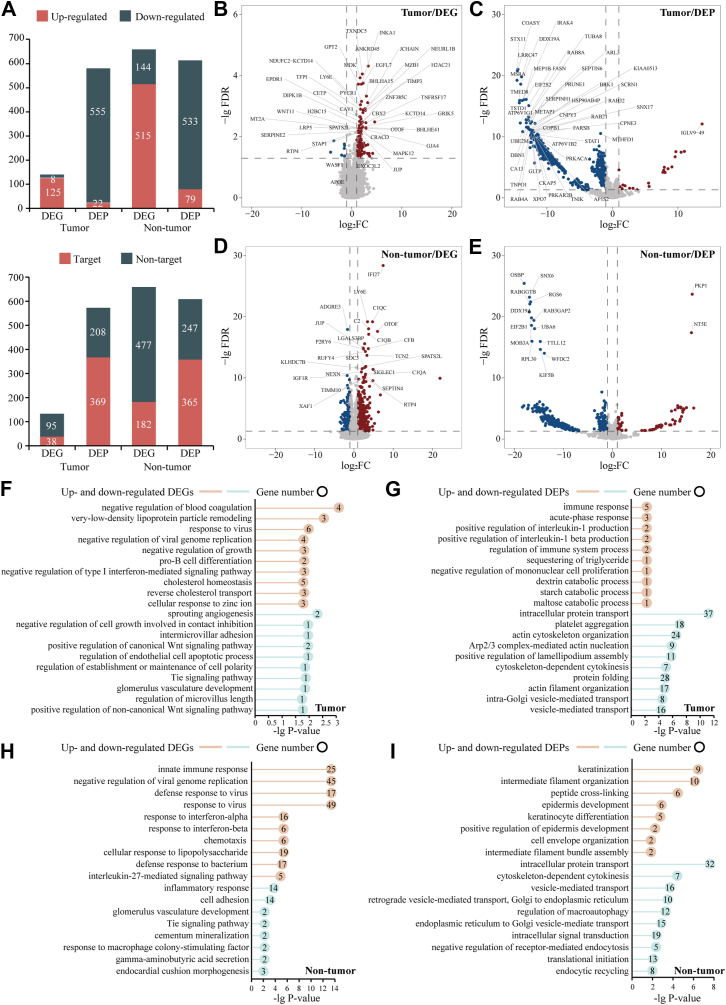
**Overview and functional enrichments of differentially expressed genes and proteins of patients having COVID-19 with and without hematological tumors post-SARS-CoV-2 infection.***A*, distribution of up- and down-regulated DEGs and DEPs in tumor patients and non-tumor individuals post-SARS-CoV-2 infection, and corresponding distribution of SARS-CoV-2 targets/nontargets. *B–E*, up- and down-regulated DEGs/DEPs post-SARS-CoV-2 infection in hematological tumor patients (*B* and *C*) and non-tumor individuals (*D* and *E*). *F–I*, GO biological processes enriched in up- and down-regulated DEGs/DEPs for hematological tumor patients (*F* and *G*) and non-tumor individuals (*H* and *I*). Bar length represents –lg (adjusted *p*-value). Terms with n < 5 genes require cautious interpretation.

To elucidate the functional impact of SARS-CoV-2 infection, we performed functional GO and KEGG pathway enrichment analyses on the identified DEGs and DEPs post-SARS-CoV-2 infection. Most DEGs were up-regulated post-infection, while DEPs were predominantly down-regulated (Fig. 2*A*). Notably, the proportion of SARS-CoV-2 interacting proteins was significantly higher in DEPs compared to DEGs (Fisher exact test, *p*-value <0.05), suggesting that SARS-CoV-2 preferentially modulates host responses at the proteomic rather than the transcriptomic level. Enriched functions and pathways included immune response, cytokine-mediated signaling, and pathogen response, with consistent trends between DEGs and DEPs (Fig. 2, *B*–*I* and Supplemental Tables S6 and S7). For instance, up-regulated DEGs in tumor patients were enriched in processes such as “negative regulation of blood coagulation” (*e.g.*, APOE, SERPINE1, SERPINE2, and TFPI), while down-regulated DEPs were associated with “platelet aggregation” (*e.g.*, CLIC1, HSPB1, and ITGB3) (Fig. 2, *F*–*G*). Interestingly, we also observed several functional differences between DEGs and DEPs post-SARS-CoV-2 infection. In both tumor patients and non-tumor individuals, “translation initiation” (*e.g.*, DDX3X, EIF2S1, and EIF2S2) was significantly enriched in down-regulated DEPs (Fig. 2, *G* and *I*), suggesting potential suppression of protein synthesis or dysregulation at the post-transcriptional level following SARS-CoV-2 infection. This observation may explain the higher number of up-regulated DEGs and down-regulated DEPs, highlighting a decoupling of transcriptional and translational responses during infection. Furthermore, biological processes such as “response to virus” and “negative regulation of viral genome replication” were significantly enriched with DEGs but not with DEPs (Fig. 2, *F* and *H*), indicating that SARS-CoV-2 exerts a more direct influence on transcriptional regulation than on protein expression. Additionally, down-regulated DEGs were enriched in vascular development-related processes, such as the “Tie signaling pathway” and “glomerulus vasculature development” (Fig. 2, *F* and *H*), whereas DEPs were predominantly associated with actin-related processes, including “actin cytoskeleton organization”, “Arp2/3 complex-mediated actin nucleation”, and “actin filament organization” (Fig. 2, *G* and *I*). These findings highlight both concordance and divergence between transcriptomic and proteomic data, providing deeper insights into SARS-CoV-2-induced immune dysregulation.

**Fig. 3:**
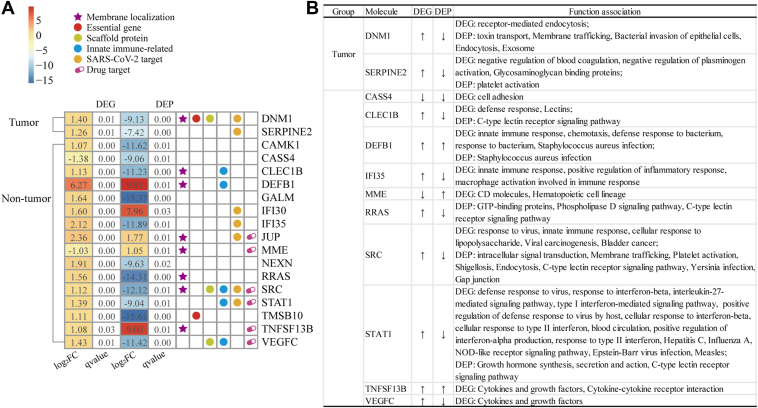
**The overlapping between differentially expressed genes and proteins of COVID-19 patients with and without hematological tumors post-SARS-CoV-2 infection and the corresponding functions involved.***A*, overlaps of DEGs and DEPs in hematological tumor patients and non-tumor individuals. q = 0.00 denotes q < 0.001. *B*, enriched functional terms involved in the key molecules. *Up/down arrows* indicate up-/downregulation of the DEGs and DEPs.

We identified 2 and 16 overlaps between DEGs and DEPs in patients with tumor and non-tumor individuals, respectively (Fig. 3*A*). The key molecules exhibited discordant regulation (*e.g.*, STAT1↑DEG↓DEP; DNM1↑DEG↓DEP), while several non-tumor cases showed concordance (DEFB1↑↑; TNFSF13B↑↑). Functional analysis revealed that discordantly upregulated DEGs were specifically linked to antiviral or immune processes (*e.g.*, STAT1: defense response to virus; SRC: response to virus), while discordantly downregulated DEPs were associated with signaling pathways and infection responses (*e.g.*, STAT1↓DEP: chemokine signaling; CLEC1B↓DEP: C-type lectin receptor signaling; SRC↓DEP: VEGF/chemokine signaling) (Fig. 3*B*, Supplemental Tables S6 and S7). This transcriptional-translational decoupling suggests that SARS-CoV-2 may disrupt host defenses through post-transcriptional suppression of critical signaling effectors—consistent with viral strategies to hijack host machinery (*e.g.*, via NSP1) (58). Our data indicate that direct viral processes and immune responses were primarily modulated transcriptionally (*e.g.*, antiviral DEGs: STAT1, IFI35, CLEC1B), while downstream signaling and infection-related pathways (*e.g.*, toxin transport, C-type lectin signaling, chemokine signaling, intracellular signal transduction) were disrupted at the proteome level (*e.g.*, DEPs of STAT1, SRC, RRAS, DNM1). While limited in number, these discordantly regulated molecules pinpoint key viral evasion mechanisms (*e.g.*, suppression of STAT1 protein despite transcriptional upregulation) and prioritize targets for therapeutic intervention (*e.g.*, DNM1 in endocytosis; SERPINE2 in coagulation dysregulation).

### Network Topology and Functional Role Analysis of DEGs and DEPs

**Fig. 4:**
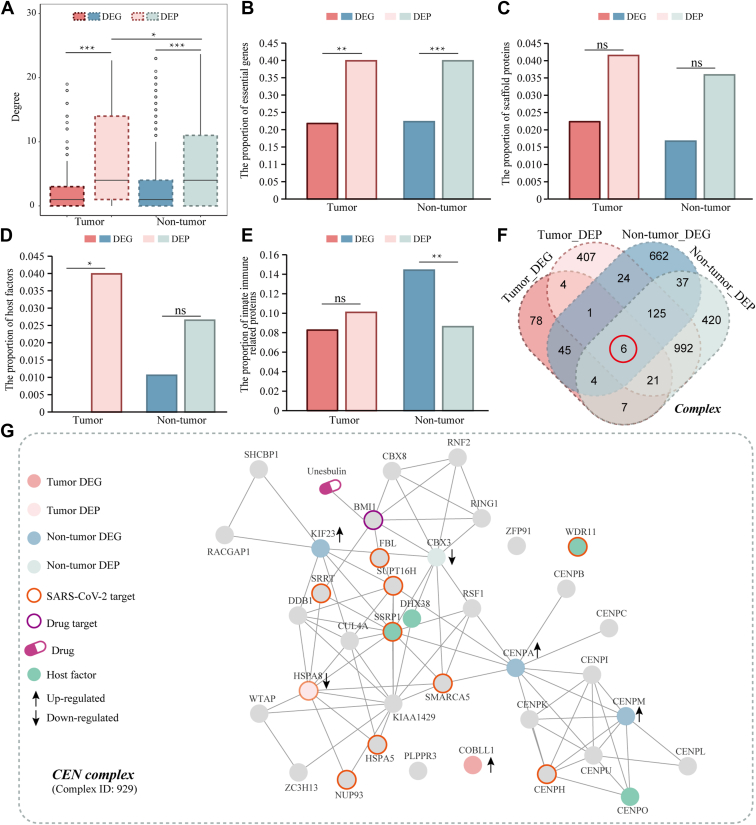
**Comparison of network and functional complex characteristics between differentially expressed genes and proteins of hematological tumor patients/non-tumor individuals.***A*, Degree distributions of DEGs and DEPs. Proportions of essential genes (*B*), scaffold proteins (*C*), host factors (*D*) and innate immune-related proteins (*E*) of DEGs and DEPs. *F*, complex distribution in DEGs/DEPs of hematological tumor patients/non-tumor individuals post-SARS-CoV-2 infection. *G*, specific complex case and corresponding network functional annotations, i.e., the CEN complex covering both DEGs/DEPs in tumor and non-tumor individuals.

In terms of network topological features, viruses typically hijack human host machines to perform their own life activities by targeting hub proteins (26, 59). Using a network of 80,258 human PPIs, we calculated the number of interaction partners of each DEG and DEP (i.e., degree) in this PPI network. We found that DEPs exhibited significantly higher degrees than DEGs (Wilcoxon rank sum test, *p*-values < 0.001; Fig. 4*A*) in both tumor patients and non-tumor individuals, indicating their central role in SARS-CoV-2-induced perturbations. Furthermore, DEPs in tumor patients had higher degrees than in non-tumor individuals (Wilcoxon rank sum test, *p*-value < 0.05; Fig. 4*A*), suggesting that hub proteins play a critical role in tumor-specific responses to infection.

We further compared the functional roles of DEGs and DEPs by assessing the proportions of essential genes, scaffold proteins, host factors, and innate immune-related proteins (Fig. 4, *B*–*E*). DEPs showed a significantly higher proportion of essential genes compared to DEGs in both tumor and non-tumor individuals (Fig. 4*B*), indicating that essential cellular functions are more disrupted at the proteomic level. While scaffold protein proportions were similar between DEGs and DEPs (Fig. 4*C*), host factors were more enriched in DEPs of tumor patients (Fig. 4*D*), suggesting that SARS-CoV-2 preferentially targets host factors at the protein level to facilitate infection. Interestingly, innate immune-related genes/proteins were more enriched in DEGs than DEPs in non-tumor individuals (Fig. 4*E*). Such observation suggested that innate immune responses were predominantly at the transcriptional level rather than the proteomic level.

To explore functional complexes, we mapped DEGs and DEPs onto human protein complexes to identify differentially expressed complexes (DECs) (Fig. 4*F* and Supplemental Table S8). Focusing on the six complex overlaps of DEGs/DEPs in tumor patients and non-tumor individuals, we considered the centromer chromatin complex (CEN complex), as an instance to perform complex network-based drug repositioning (Fig. 4*G*). By integrating human PPIs, human-SARS-CoV-2 PPIs, drug-protein interactions, and drug-disease relationships, we obtained a comprehensive network containing DEGs/DEPs to identify potential drug targets. For example, BMI1, a known drug target interacting with the non-tumor DEP CBX3, and SRRT, an interactor of the tumor DEP HSPA8, were highlighted. Unsebulin, a BMI1 inhibitor, emerged as a potential repurposing candidate. Other potential drug targets included HSPA8 (membrane location), NUP93, HSPA5, SRRT, SSRP1, DHX38, SMARCA5, SUPT16H, FBL, CENPH, CENPO, and WDR11, which are implicated in SARS-CoV-2 infection and host response.

### Comparative Analysis of Molecular Responses in Tumor *versus* Non-Tumor Patients With COVID-19

**Fig. 5:**
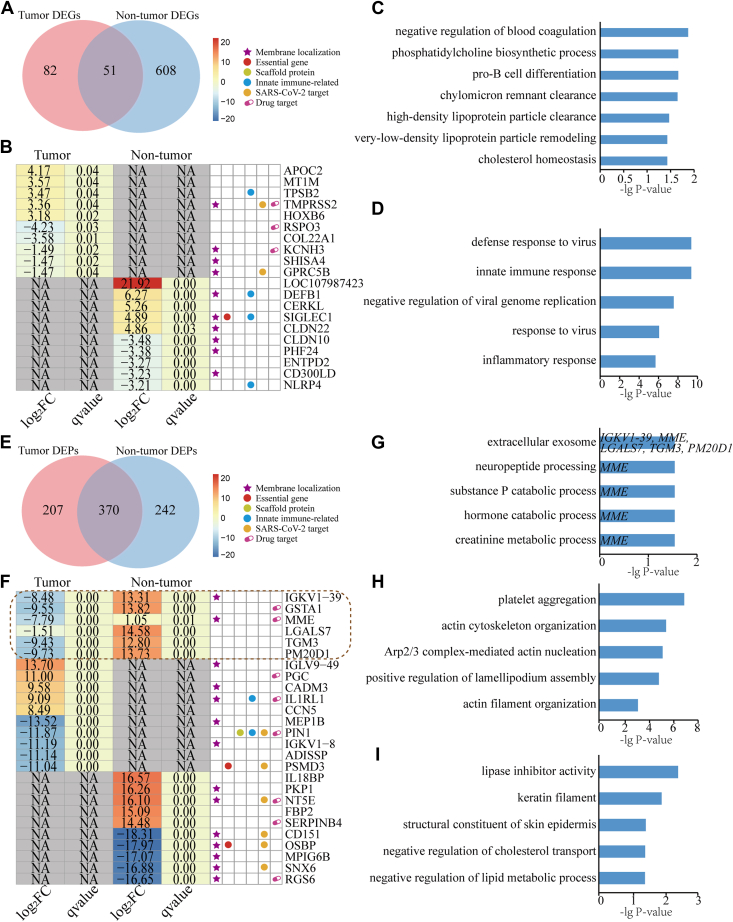
**Multi-omics analysis of dysregulated genes and proteins in COVID-19 patients with/without hematological tumors.***A*, *Venn diagrams* of differentially expressed genes (DEGs) between tumor patients (n = 133) and non-tumor individuals (n = 659). Intersections denote shared DEGs; non-overlapping regions indicate tumor-specific DEGs (*pink*) and non-tumor-specific DEGs (*blue*). *B*, Heatmap of top 5 uniquely upregulated/downregulated DEGs per group. Color scale: Log_2_ fold change (*red*: upregulation; *blue*: downregulation). q = 0.00 denotes q < 0.001. NA, tumor/non-tumor-specific DEGs. *C* and *D*, functional enrichment of tumor-specific DEGs (*C*) and non-tumor-specific DEGs (*D*). *Top* terms ranked by adjusted *p*-value (Benjamini-Hochberg correction); *Bar length* indicates −lg (adjusted *p*-value). *E*, *Venn diagrams* of differentially expressed proteins (DEPs) (tumor: n = 577; non-tumor: n = 612). *F*, heatmap of six oppositely regulated DEPs between groups and top 5 uniquely upregulated/downregulated DEPs per group. Color scale and NA as in (*B*). *G*, subcellular localization of the five reversed DEPs localize to extracellular exosomes (UniProt database). *H* and *I*, functional enrichment of tumor-specific DEPs (*H*) and non-tumor-specific DEPs (*I*), highlighting key functional differences in platelet aggregation (tumor) *versus* epidermal differentiation (non-tumor).

To elucidate the molecular basis for the increased vulnerability of hematological tumor patients to COVID-19, we performed comprehensive comparative analyses of transcriptomic and proteomic profiles between tumor and non-tumor cohorts. Our analysis identified 82 unique DEGs in tumor patients compared to 608 in non-tumor individuals (Fig. 5*A*). Notably, the top differentially regulated genes in tumor patients were enriched for genes involved in immune regulation (*e.g.*, SOX4, FLT3) and cellular metabolism (*e.g.*, APOC2, APOE), while non-tumor individuals showed predominant upregulation of antiviral defense genes (*e.g.*, STAT1, HERC5) (Fig. 5*B* and Supplemental Table S9).

Pathway analysis demonstrated fundamentally different host response mechanisms between the two groups. Tumor patients displayed significant dysregulation across multiple biological processes, including impaired immune cell differentiation pathways (pro-B cell differentiation), disrupted lipid metabolic processes (phosphatidylcholine biosynthetic process, lipoprotein remodeling), and abnormal coagulation function (negative regulation of blood coagulation) (Fig. 5*C*). In contrast, non-tumor individuals mounted robust innate immune responses characterized by effective antiviral defense mechanisms and properly regulated inflammatory responses (Fig. 5*D*).

The proteomic findings further reinforced these differences, identifying 207 unique DEPs in tumor patients *versus* 242 in non-tumor cases (Fig. 5*E*). A particularly noteworthy discovery was the identification of six proteins that showed completely opposite regulation patterns between the groups (Fig. 5*F*). Out of these proteins, five are associated with extracellular exosomes, and play critical roles in glutathione metabolism and neuropeptide catabolism (Fig. 5*G*). Among them, MME emerged as especially significant due to its established role in immune regulation.

Tumor-specific proteomic alterations were particularly evident in pathways related to platelet function and cytoskeletal organization, providing molecular correlates for the coagulation abnormalities frequently observed in COVID-19 patients with hematological malignancies (Fig. 5*H*). Conversely, non-tumor individuals showed preserved activation of epithelial barrier maintenance pathways, suggesting intact first-line defense mechanisms against viral infection (Fig. 5*I*).

These multi-omics findings collectively provide a mechanistic explanation for the clinical vulnerability of hematological tumor patients to severe COVID-19 outcomes. The data highlight three key compromised systems: (i) deficient antiviral immune responses, (ii) dysregulated coagulation and metabolic pathways, and (iii) impaired extracellular communication through exosomes. The comprehensive datasets from these analyses are available in Supplemental Table S9.

### Network Module Analysis and Drug Repositioning

**Fig. 6:**
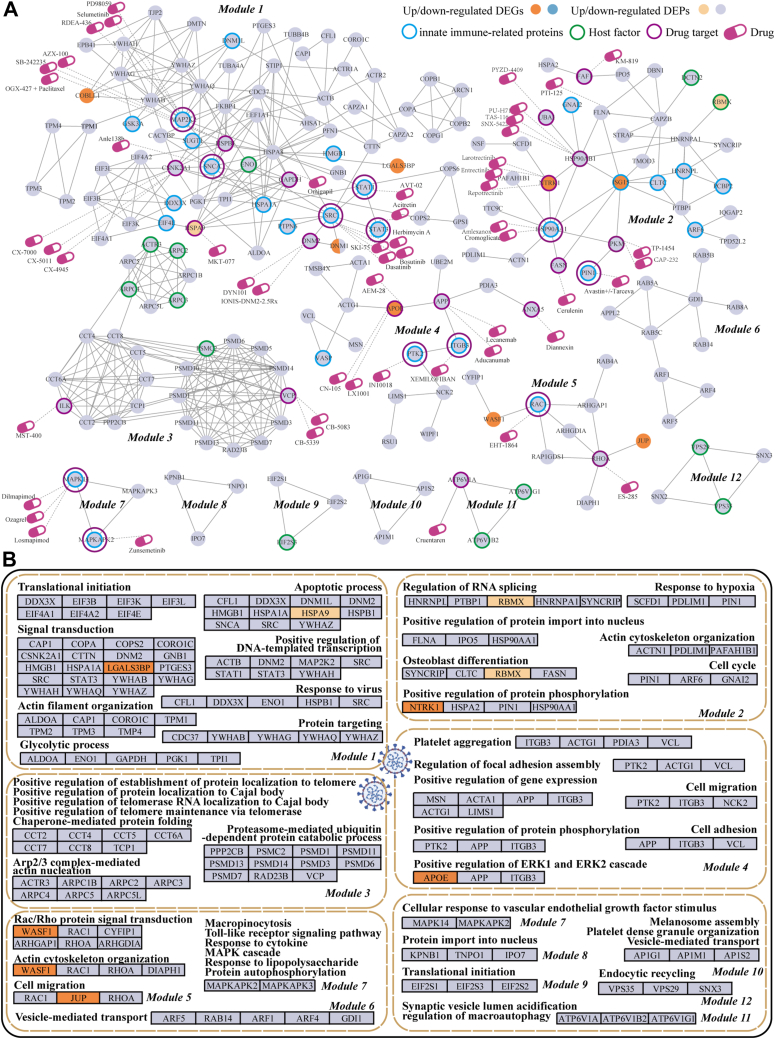
**Identified network modules of DEGs/DEPs of COVID-19 patients with hematological tumors post-SARS-CoV-2 infection.***A*, Network topological modules of up/down-regulated DEGs and DEPs and their functional gene set annotations as well as drug repositioning. *B*, significantly enriched GO terms of each network module and corresponding genes/proteins.

To explore the network and functional associations of DEGs and DEPs in hematological tumor patients, we integrated DEGs/DEPs with protein interactions to identify 12 functional modules (Fig. 6*A*), annotated with immune properties and drug targets. The modules were involved in diverse biological processes, such as translational initiation, apoptotic process, protein phosphorylation, RNA splicing, telomerase maintenance, ubiquitin-dependent protein catabolic process, platelet aggregation, cell adhesion, actin cytoskeleton organization, and vesicle-mediated transport (Fig. 6*B* and Supplemental Table S10).

The COBLL1-YWHA immune regulatory axis featured upregulated tumor-specific DEG COBLL1 and down-regulated YWHA proteins (YWHAB/YWHAG/YWHAZ↓), which are RNA-binding proteins (60) involved in viral response and signal transduction. This suggests transcriptome-proteome correlation in immune regulation. The HSPA9-SRC-STAT1 immune-cytoskeletal axis featured upregulated HSPA9, down-regulated SRC and STAT1 (core DEPs), which may suppress dynamin-mediated endocytosis (DNM1/DNM2↓) and impaire viral clearance via the bacterial invasion pathway (KEGG: has05100). Through drug repositioning, we identified potential therapeutic targets with established drugs for other diseases, including HSPA9 (originally targeted in solid tumors; *e.g.*, MKT-077), SRC (hematological tumor; *e.g.*, dasatinib/bosutinib), DNM2 (congenital myopathy; *e.g.*, DYN101), STAT1/STAT3 (immune disorders; *e.g.*, AVT-02/acitretin), GAPDH (muscular dystrophy; *e.g.*, omigapil), SNCA (Alzheimer; *e.g.*, Anle138b), and CSNK2A1 (viral infection; *e.g.*, CX-7000) (Fig. 6*A*).

In module 2, the upregulated DEG NTRK1 and downregulated DEPs HSP90AB1/HSP90AA1 (known drug targets) were part of the PI3K pathway. In particular, HSP90AA1/HSP90AB1 also interact with the up-regulated DEG ISG15, an innate immune-related protein linked to interferon signaling, suggesting its role in amplifying inflammatory responses. Furthermore, the only up-regulated DEP RBMX (a host factor) interacts with HNRNPA1, both of which are components of the spliceosome pathway, indicating their potential role in RNA splicing regulation post-SARS-CoV-2 infection (Fig. 6, *A* and *B*). Such observations indicated potential associations between these DEGs and DEPs and highlighted several known or potential drug targets. In module 5, the up-regulated DEGs WASF1 and JUP interact with known drug targets RAC1 (Alzheimer disease) and RHOA (solid tumor), respectively (Fig. 6*A*), and were enriched in biological processes such as “Rac/Rho protein signal transduction”, “actin cytoskeleton organization”, and “cell migration” (Fig. 6*B*). In particular, RAC1, WASF1, and RHOA were involved in pathways related to actin generation, such as “regulation of actin cytoskeleton” (KEGG: map04810; RHOA→RAC→WASF1→Arp2/3→actin), highlighting the significant impact of SARS-CoV-2 infection on actin dynamics and cytoskeletal organization.

In our previous work, we identified APOE as a tumor-specific up-regulated DEG involved in blood coagulation and lipoprotein transport, suggesting a potential impact of SARS-CoV-2 on lipid metabolism (32). Here, we further found that APOE interacts with APP in module 4 (Fig. 6*A*). Both proteins are associated with the Alzheimer's disease pathway and the biological process “positive regulation of ERK1 and ERK2 cascade” (Fig. 6*B*). The differential effects of APOE2, APOE3, and APOE4 on APP transcription and Aβ secretion (61) suggest that APOE and APP may synergistically affect the nervous system following SARS-CoV-2 infection, in addition to their roles in cholesterol metabolism. Through drug repositioning, we identified potential therapeutic targets with established drugs for other diseases, including APOE (hypercholesterolemia, hyperlipidemia, and Alzheimer disease; *e.g.*, AEM-28), APP (solid tumor and Alzheimer disease; *e.g.*, lecanemab), PTK2 (solid tumor and pulmonary arterial hypertension; *e.g.*, IN10018), ITGB3 (chronic arterial occlusive disease; *e.g.*, XEMILOFIBAN), and ANXA5 (myocardial reperfusion injury; *e.g.*, diannexin) (Fig. 6*A*).

### Clinical and Omics Analysis of Patients Having Hematological Tumor With Different Infection Outcomes

**Fig. 7:**
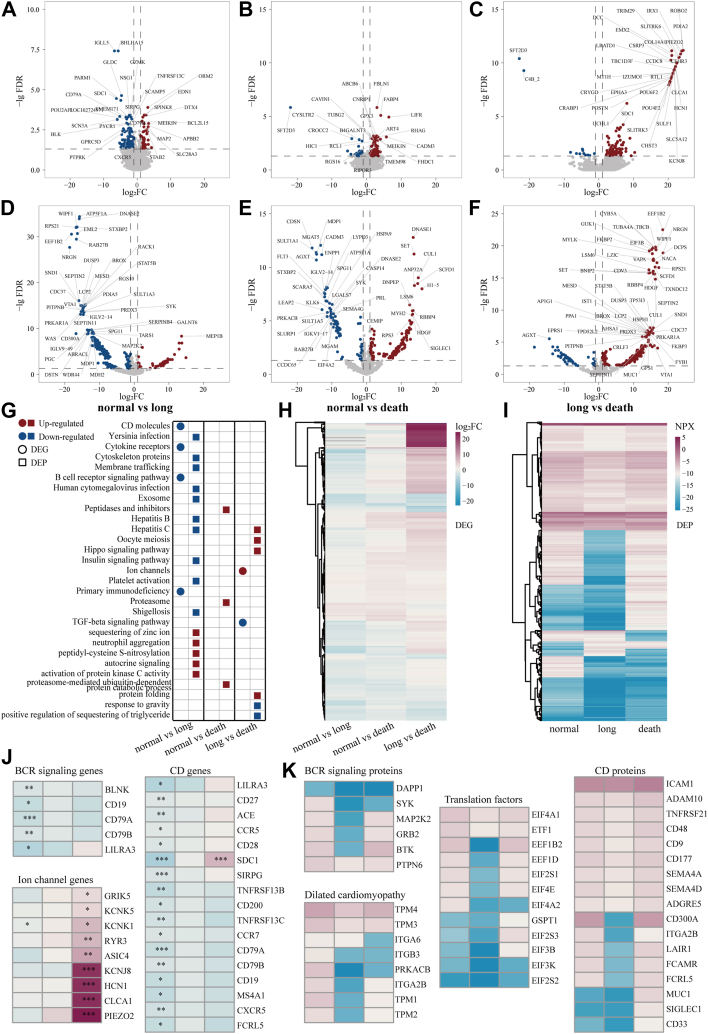
**DEGs and DEPs between multiple infection status of hematological tumor patients during the acute infection phase.** Volcano plots highlight DEGs (*A*–*C*) and DEPs (*D*–*F*) between multiple infection status including normal infection, long infection, and acute death of patients with hematological tumors. *G*, enriched GO biological processes and KEGG pathways of up- and down-regulated DEGs and DEPs. *H*, heatmap shows clusters based on log_2_FC of DEGs between different infection groups. *I*, heatmap shows clusters based on normalized protein expression (NPX) of DEPs between different infection groups. *J*, heatmaps show the log_2_FC values of select BCR, CD, and ion channel genes from (*H*). *K*, heatmaps show the NPX values of select BCR, CD, translational, and dilated cardiomyopathy-associated proteins from (*I*). NPX represents quantile-normalized, log_2_-transformed protein abundance calculated by OmicsBean where higher values indicate higher protein abundance.

**Fig. 8:**
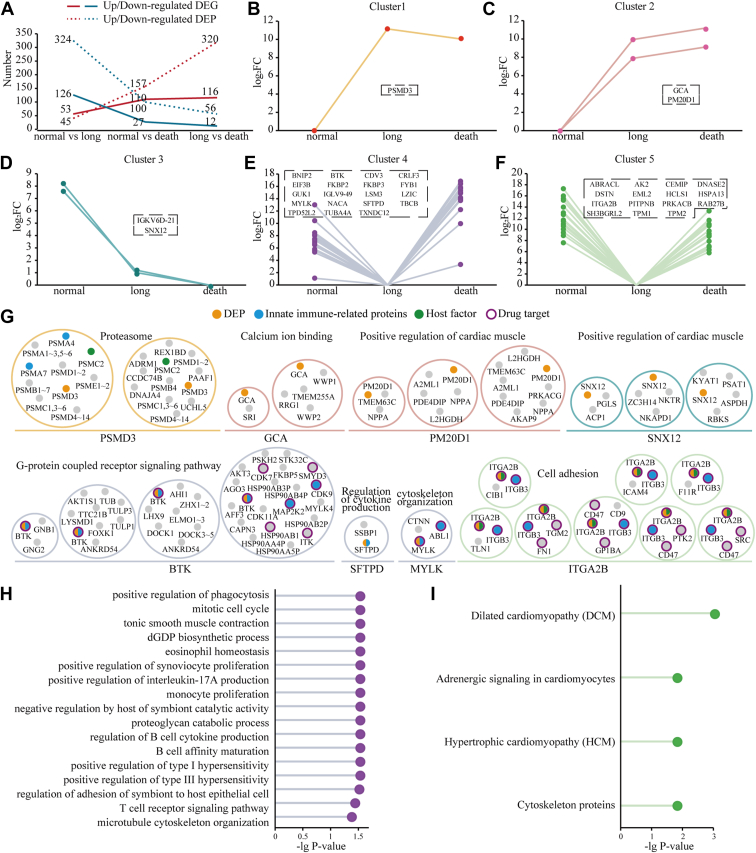
**Clustering analysis and functional characterization of DEPs across infection outcomes in hematological tumor patients.***A*, the total counts of up- and down-regulated DEGs and DEPs between infection status groups (normal infection, long infection, and acute death). *B–F*, clusters 1 to 5 represent manually grouped DEPs with significant differential expression (adjusted *p*-value <0.05, |log_2_FC|>1) in all three pairwise comparisons (normal *versus* long, normal *versus* death, long *versus* death). *G*, specific differentially expressed complexes involved in the five clusters. Significantly enriched functional terms of the protein sets of cluster 4 (*H*) and cluster 5 (*I*).

To elucidate the differences in infection mechanisms among hematological tumor patients with varying clinical outcomes, we analyzed transcriptomic and proteomic changes in mid-infection samples (collected ≤3 days post-symptom onset), stratifying patients into three outcome-based groups (i.e., normal infection, long infection, and acute death) (Fig. 7, *A*–*F*). We identified 179, 137, and 128 DEGs in the comparisons of “normal *versus* long”, “normal *versus* death”, and “long *versus* death”, respectively (Fig. 7, *A*–*C*). Correspondingly, 369, 257, and 376 DEPs were identified in these groups (Fig. 7, *D*–*F*). We observed consistent trends in DEGs and DEPs, in which the numbers of up-regulated DEGs/DEPs gradually increased, while the numbers of down-regulated DEGs/DEPs gradually decreased from “normal *versus* long” to “normal *versus* death” and finally to “long *versus* death” (Fig. 8*A*). Such observations indicated that most DEGs/DEPs were highly expressed in acutely deceased patients compared to patients with long infection, and more highly expressed in patients with normal infection than in those with long infection.

Pathway enrichment analysis revealed distinct pathway dysregulation patterns associated with different COVID-19 outcomes (Fig. 7*G*). Compared to patients with normal infection, patients with long COVID exhibited downregulation of multiple pathways, particularly those related to membrane trafficking, platelet activation, cytoskeleton organization, and various infections (*e.g.*, *Yersinia*, Shigellosis, Hepatitis B/C, HCMV), as well as insulin signaling and exosome function. Conversely, acutely deceased patients (compared to normal infection) showed upregulation of pathways involved in proteasome-mediated ubiquitin-dependent protein catabolism, proteasome activity, and peptidase function. Furthermore, when compared directly to patients with long COVID, deceased patients displayed upregulation of pathways including protein folding, Hepatitis C, oocyte meiosis, and Hippo signaling, alongside downregulation of pathways involved in response to gravity and positive regulation of triglyceride sequestration.

Crucially, adaptive immunity pathways were impaired in long COVID; specifically, the B cell receptor (BCR) signaling pathway was significantly downregulated in patients with long COVID relative to those with normal infection (Fig. 7*G*). Clustering analysis of DEGs and DEPs identified key functional genes/proteins associated with COVID-19 severity (Fig. 7, *H* and *I*). Consistent with the pathway findings, genes and proteins involved in BCR signaling and CD molecules were downregulated in long COVID patients (Fig. 7J and K). Additionally, the expression of ion channel genes was increased in acutely deceased patients compared to long COVID patients (Fig. 7J).

Clinical characterization of the three groups (Table 1 and Supplemental Table S1) corroborated these molecular findings. Compared to patients with normal infection, patients with long COVID showed significantly prolonged thrombin time (TT), indicating coagulation abnormalities (Wilcoxon rank sum test, *p*-value <0.05; Table 1). Acutely deceased patients exhibited more extensive dysregulation, including abnormalities in additional coagulation parameters [prothrombin time (PT) and international normalized ratio (INR)] and immune-related biomarkers [interleukin-6 (IL-6), procalcitonin (PCT), and albumin (ALB)] (Wilcoxon rank sum test, *p*-values <0.05; Table 1). These clinical observations align with the molecular data, indicating significant coagulation dysfunction in long COVID and deceased patients, with deceased patients also demonstrating profound immune and systemic disturbances.

In our previous work (32), we identified overlapping DEGs between any two groups of “normal *versus* long”, “normal *versus* death”, and “long *versus* death”, including key genes such as APOE, FKBP10, SFT2D3, PYCR1, UCHL1, CSGALNACT1, DCBLD1, and PCBP3. Here, we extended this analysis to the proteome level, identifying five clusters of DEPs across all three groups (Fig. 8, *B*–*F*). Subsequently, we mapped human protein complexes onto these DEPs in the five clusters to identify DECs (Fig. 8*G*). In cluster 1, we found that the proteasome complex involving DEP PSMD3 was significantly enriched in long infection patients, suggesting higher proteasome activity to maintain protein homeostasis compared to normal infection and deceased patients (Fig. 8, *B* and *G*). Key components of this complex, including innate immune-related proteins (PSMA4 and PSMA7) and a host factor (PSMC2), were identified as potential drug targets. Furthermore, cluster 2 (death > long > normal) and cluster 3 (death < long < normal) showed significantly high and low expression of DEPs in acute death and long infection patients, respectively (Fig. 8, *C* and *D*). In particular, the DEPs in these two clusters were both involved in complexes associated with the biological process “positive regulation of cardiac muscle” (Fig. 8*G*), indicating severe cardiac muscle impairment in deceased and long infection patients. Cluster 4 (long < normal < death) and cluster 5 (long < death < normal) showed significantly low expression of DEPs in long infection patients (Fig. 8, *E* and *F*). Furthermore, cluster 4 was enriched in biological processes such as receptor signaling, cytokine production, and interleukin production, while cluster 5 was associated with cardiomyopathy, adrenergic signaling in cardiomyocytes, and actin filament organization (Fig. 8, *H* and *I* and Supplemental Table S11). These observations suggested that long COVID patients experience suppressed immune responses and impaired regulation of actin filaments and heart rate. Moreover, we identified several known and potential drug targets within the DECs (Fig. 8*G*), including BTK, ITK, CDK9, ABL1, MYLK, FN1, and CD47, providing valuable clues for drug repositioning to treat long COVID and its associated complications.

## Discussion

The host response to SARS-CoV-2 infection is a complex interplay of innate and adaptive immune mechanisms, where immune activation must be precisely balanced to achieve viral clearance while preventing excessive tissue damage (43). Our comprehensive multi-omics analysis of hematological tumor patients and non-tumor individuals reveals both distinct and overlapping patterns of transcriptomic (DEG) and proteomic (DEP) regulation during SARS-CoV-2 infection, providing new insights into the molecular basis of COVID-19 pathogenesis.

Our findings align with and extend prior COVID-19 proteomic studies. Consistent with reports of coagulation dysfunction in severe COVID-19 (21), we observed down-regulated DEPs (*e.g.*, CLIC1, ITGB3) in platelet aggregation pathways in tumor patients. However, our study uniquely reveals that DEPs in hematological tumors exhibit significantly higher network centrality (degree) than in non-tumor individuals (Fig. 4*A*), suggesting that hub proteins are preferentially dysregulated in immunocompromised hosts. This finding contrasts with general COVID-19 cohorts where proteomic changes primarily reflect inflammation (22), highlighting tumor-specific vulnerability mechanisms. Additionally, while long COVID proteomics have been linked to complement dysregulation (28), we identified immunosuppressive signatures (*e.g.*, suppressed BCR signaling) specific to hematological tumor patients with prolonged infection – a population excluded from most prior studies.

While previous multi-omics studies have characterized immune dysregulation in COVID-19 (22, 27, 41, 62), our comparative analysis of transcriptional and translational responses reveals three notable features requiring cautious interpretation. First, the limited overlap between DEGs and DEPs suggests potential post-transcriptional and post-translational regulatory mechanisms, though serum protein dynamics may originate from multiple biological sources beyond translation control. Specifically, DEP alterations could reflect integrated effects of cell death events, shifts in immune cell population abundance, and altered protein secretion patterns across diverse tissues—particularly given that serum proteins integrate contributions from broader biological systems compared to whole blood-derived transcriptional profiles. Second, we observed widespread upregulation of DEGs (*e.g.*, antiviral pathways) alongside downregulation of DEPs (*e.g.*, translation initiation factors), suggesting post-transcriptional repression—a phenomenon increasingly recognized in SARS-CoV-2 biology. Notably, viral proteins such as NSP1 act as ribosome gatekeepers, selectively inhibiting host mRNA translation while promoting viral protein synthesis (58). Such an observation aligns with our findings of downregulated DEPs (*e.g.*, EIF2S1, EIF2S2) in translation-related pathways, which may reflect viral hijacking of ribosomal machinery rather than purely host-driven regulation. For further support, SARS-CoV-2 disrupts splicing, translation, and protein trafficking to suppress host defenses (63). Third, our proteomic analysis revealed significant enrichment of differentially expressed proteins (DEPs) in key biological processes including “actin cytoskeleton organization” and “protein kinase activity”, both of which are commonly exploited by viruses to facilitate their replication. The discordance between upregulated innate immune DEGs (*e.g.*, STAT1) and their downregulated protein products underscores viral interference at translational checkpoints, consistent with reports that SARS-CoV-2 uses a multipronged strategy to impede host protein synthesis (64).

Our stratification of infection outcomes revealed distinct molecular signatures between long COVID and acute death cases. Acute deaths exhibited hyperactivated immune signatures, whereas long COVID patients showed persistent suppression of adaptive immune pathways (*e.g.*, BCR signaling, HLA class II expression), potentially explaining their prolonged symptomatology. These observations corroborate previous findings of immune exhaustion in severe COVID-19 (22, 65) while extending them to hematological malignancies. Phan *et al*. demonstrated that several age-related immune impairments (including compromised T/B cell immunity and MHC antigen presentation) likely promote SARS-CoV-2 persistence at the transcriptional, proteomic and cellular levels (43), leading to delayed viral clearance to facilitate the evolution of SARS-CoV-2 variants (66, 67). Notably, long COVID immunosuppression (*e.g.*, BCR downregulation) was exclusive to hematological tumor patients, contrasting with non-tumor individuals who uniformly resolved infection. Conversely, acute deaths displayed immuno-activation patterns consistent with dysregulated antigen processing/presentation and elevated inflammatory cytokines (68) — features that may reflect failed viral containment. Together, these observations highlight the spectrum of immune dysregulation in COVID-19 outcomes, from chronic immunosuppression in long COVID to hyperinflammatory responses in acute death cases.

By integrating DEGs and DEPs with human protein complexes and network-based drug repositioning, we identified potential therapeutic targets, such as HSPA8, SRC, and STAT1, as well as complex modules like APOE and APP, but their efficacy may depend on circumventing viral interference. For instance, *STAT1* downregulation at the protein level—despite transcriptional upregulation—could reflect viral evasion of interferon responses, a strategy documented in SARS-CoV-2-infected cells. Future therapies might combine antiviral agents (*e.g.*, NSP1 inhibitors) with immunomodulators to restore translational balance. These targets offer promising avenues for treating both acute COVID-19 and long-term sequelae. Notably, our approach diverges from prior COVID-19 drug studies that prioritized single targets (*e.g.*, IL-6, JAK-STAT) by instead mapping dysregulated proteins onto functional complexes to identify modular targets (*e.g.*, HSPA8 in the CEN complex; APOE-APP in Alzheimer's pathway). This approach uncovered context-specific candidates like Unsebulin (targeting BMI1-CBX3) for tumor-related COVID—a strategy not explored in prior blood proteomic works.

While our study provides valuable insights into host molecular responses to SARS-CoV-2 infection in hematological tumor patients and non-tumor individuals, several limitations should be noted. First, the modest sample size may affect the generalizability of our findings. Second, although we integrated transcriptomic and proteomic data, additional omics layers (*e.g.*, metabolomics, epigenomics, single-cell sequencing) could further clarify the underlying mechanisms. Third, while we identified potential therapeutic targets, functional validation is needed to confirm their roles in infection and therapeutic potential. Importantly, our proteomic analysis focused on host proteins and did not directly assess viral proteins due to technical challenges: serum viral proteins often fall below detection limits in untargeted mass spectrometry without prior enrichment (*e.g.*, immuno-depletion or targeted assays). While RT-PCR confirmed active infection, future studies could combine viral proteomics with host multi-omics to better understand persistence mechanisms, as demonstrated in recent studies integrating viral proteomic dynamics (13, 69, 70). Additionally, the comparison between whole blood transcriptomics and serum proteomics requires careful interpretation. While whole blood RNA provides cellular transcriptional insights, serum proteins reflect integrated contributions from multiple tissues and cell types, influenced by processes such as protein secretion, cell death, and tissue leakage. These differences preclude direct inference of cell-type-specific translational regulation. Future studies employing matched single-cell or tissue-specific multi-omics approaches would help elucidate the cellular origins of these systemic changes. Despite these limitations, our findings advance the understanding of SARS-CoV-2-host interactions. Large-scale, functionally validated studies incorporating viral and additional omics data will further enhance the clinical relevance of these insights.

## Data Availability

Data supporting the findings of this study have been deposited in iProX (integrated proteome resources) ProteomeXchange under accession code PXD063172. The data needed to evaluate the conclusions in the paper are present in the paper and/or the Supplementary Materials. The data that support the findings of this study are available from the corresponding author upon reasonable request.

## Supplemental Data

This article contains supplemental data.

## Ethics Approval and Consent to Participate

The study (ClinicalTrials.gov, NCT05683353) was reviewed and approved by the Biomedical Research Ethics Committee of Peking University First Hospital (Approval number:2022yan600 to 002).

## Conflict of Interest

The authors declare that they do not have any conflicts of interest with the content of this article.
