# Supplementary material for: Multi-Omics Blood Atlas Reveals Host Immune Response Features of Immunocompromised Populations Following SARS-CoV-2 Infection
Source: Mol Cell Proteomics. 2025 Sep 10;24(10):101068. doi: 10.1016/j.mcpro.2025.101068 (PMC12547266; doi:10.1016/j.mcpro.2025.101068)
Supplement: Supplementary Information [file mmc1.docx]

**Supplementary information**

## Multi-omics blood atlas reveals host immune response features of immunocompromised populations following SARS-CoV-2 infection

Xiaodi Yang^1#^, Ye Shen^1#^, Bo Tang^1#^, Jialin Zhu^1^, Bingjie Wang^1^, Qingyun Wang^1^, Wenmin Tian^2^, Stefan Wuchty^3,4,5,6^, Ziding Zhang^7^, Zeyin Liang^1*^, Yujun Dong^1*^

^*^Corresponding authors: Yujun Dong, [dongy@hsc.pku.edu.cn](mailto:dongy@hsc.pku.edu.cn); Zeyin Liang, [walzyaw@163.com](mailto:walzyaw@163.com).

The supplementary material contains:

1. Supplementary Figure S1
2. Supplementary Table S1-S11


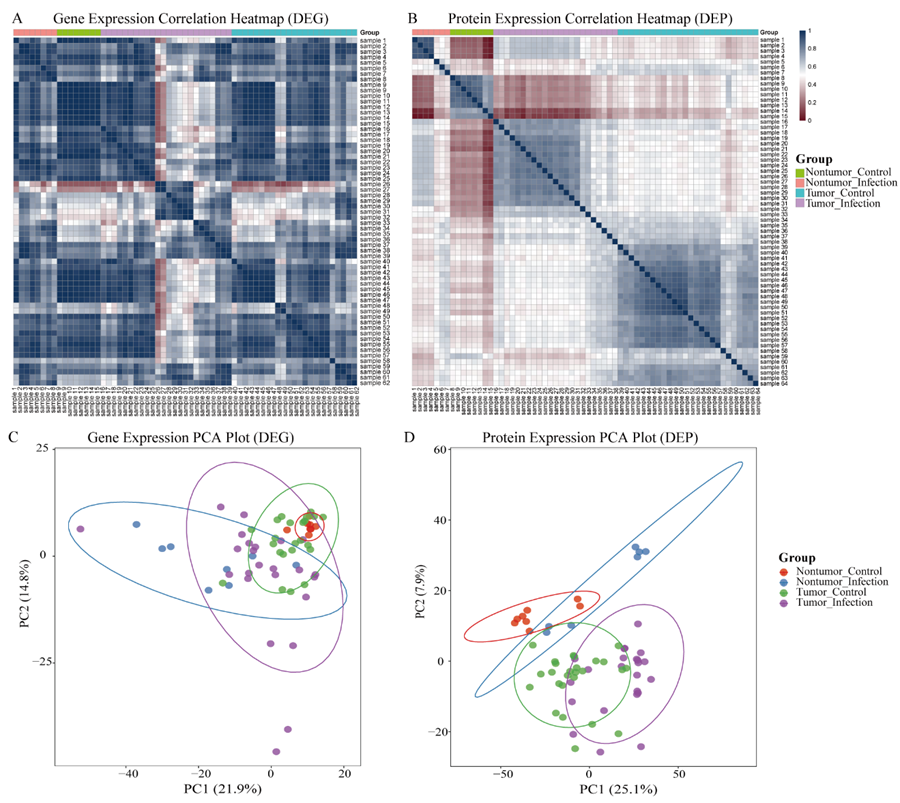


**Figure S1.** Technical validation of multi-omics data quality. (A) Inter-replicate correlation analysis of differentially expressed genes (DEGs) across whole-blood RNA-seq samples from hematological tumor and non-tumor cohorts. Heatmap colors indicate Pearson correlation coefficients (scale: 0-1). (B) Inter-replicate correlation analysis of differentially expressed proteins (DEPs) across serum proteomics samples. (C) Principal Component Analysis (PCA) of DEG expression profiles, showing separation by infection phase [mid-infection (infection)/post infection convalescence (control)] and cohort (tumor/non-tumor). (D) PCA of DEP expression profiles, demonstrating grouping by infection phase [mid-infection (infection)/post infection convalescence (control)] and cohort (tumor/non-tumor).
